# Implementation of geriatric assessment and decision support in residential care homes: facilitating and impeding factors during initial and maintenance phase

**DOI:** 10.1186/1472-6963-13-8

**Published:** 2013-01-05

**Authors:** Marijke Boorsma, Eveliene Langedijk, Dinnus HM Frijters, Giel Nijpels, Tom Elfring, Hein PJ van Hout

**Affiliations:** 1 Department of General Practice and Elderly Care Medicine, EMGO Institute for Health and Care Research, VU University Medical Center, Van der Boechorststraat 7, Amsterdam, 1081 BT, The Netherlands; 2 Business Administration, VU University Amsterdam, Amsterdam, The Netherlands

## Abstract

**Background:**

Successfully introducing and maintaining care innovations may depend on the interplay between care setting, the intervention and specific circumstances. We studied the factors influencing the introduction and maintenance of a Multidisciplinary Integrated Care model in 10 Dutch residential care homes.

**Methods:**

Facilitating and impeding factors were studied and compared at the time of introduction of the interRAI-LTCF assessment method in residential care homes as well as three years later, by surveys and semi structured interviews among nurse staff, managers, and physicians.

**Results:**

Facilitating factors at introduction were positive opinions of staff and family physicians about the changes of the process of care and the anticipated improvement of quality of care. Staff was positive about the applicability of the software to support the interRAI-LTCF assessments. Impeding factors were time constraints to complete interRAI-LTCF assessments and insufficient computer equipment.

In the maintenance phase, the positive attitude of the location manager and the perceived benefits of the care model and the interRAI-LTCF assessment method were most important. Impeding factors after 3 years remained the lack of time to complete the assessments and lack of sufficient computer equipment.

**Conclusions:**

Impeding and facilitating factors were comparable in the initial and maintenance phase. Adoption of the interRAI-LTCF assessment method depended on positive opinions of staff and management, continuing support of staff and the availability of sufficient computer equipment.

## Background

Quality of care for elderly in residential care homes is under pressure in the Netherlands as in other countries [1]. Facilities tend to be understaffed and the care complexity of residents increases while expertise of staff does not keep pace [2]. Although most care organizations want to innovate and improve their quality of care, many lack the expertise or financial resources to do so [3,4]. Family physicians are responsible for the medical care in Dutch residential care homes but do not regard themselves suited for systematic management and long-term monitoring of chronic diseases and disabilities associated with frail health [5]. Around 10% of all the elderly aged 75 or older in the Netherlands live in residential care homes. These homes were used to offer sheltered living for disabled but still relatively healthy elderly. Nowadays, due to our ageing population the characteristics of the residents of residential homes have become more and more comparable with those of nursing homes, needing complex care. These homes are publically funded and subject to governmental inspection and approval. The average size of the study residences was 46 and staff included nurse assistants, team coaches and a home manager. In some residential care homes special care wards are installed for very frail or demented persons. Systems of multidisciplinary consultations and multidisciplinary meetings are not structured nor held on a regularly base. Most family physicians do not attend those meetings. These conditions were the ingredients for the development and implementation of a new care model in a north-west region of the Netherlands. This new care model was inspired by the chronic care model and was called the Multidisciplinary Integrated Care model. The design of this study is previously reported [6].

Multidisciplinary Integrated Care comprised five elements:

1. A three-monthly geriatric multidimensional assessment of all residents. The web-based Long Term Care Facility version 9.0 of the Resident Assessment Instrument was used for this purpose [7]. The identified problem areas guide the design of an individualized care plan with the intention to improve or maintain the functional health status (Additional file 1: Figure S1).

2. The care plan was discussed with the resident, family, and family physician, and adapted to personal wishes.

3. Residents with complex care needs were scheduled at least twice a year for a multidisciplinary meeting.

4. Consultation by elderly care physician or psychologist was optional for the frailest residents with complex health care problems.

5. Data from the web based Resident Assessment Instrument was used to provide a three-monthly overview of 32 risk adjusted indicators of quality of care, which are compared to the benchmark made out of all residents of residential homes in the Netherlands using this instrument [8-11].

The effects on quality of care of this innovation were studied in a Randomised Controlled Clinical Trial in ten residential care homes in the Netherlands. The intervention homes scored better on 30 of the 32 risk indicators for quality of care and 13 of these had improved significantly [16]. Most notably, bowel incontinence decline dropped from 23 to 6%, bladder incontinence decline dropped from 46 to 15%, delirium risk dropped from 56 to 28%, pain worsening dropped from 41 to 13%. The residents in the intervention homes tended to be more satisfied about the quality of care than elderly in the control homes.

Reviews of dissemination and implementation strategies suggest that success depends on the type of care setting, type of intervention and specific circumstances [12]. People working in healthcare organizations mostly focus on their own profession. This professional identification limits the level of organizational identification and limits the willingness to collaborate across specialisations or departments, which is imperative when organisations want to work on improvements and innovation on an organisational level [13,14]. A review by Grimshaw et al. (1999) showed that obstacles to use guidelines can arise at different levels of the health care system: at the level of the patients, the individual professional, the health care team, the health care organization or the wider environment [8,15]. The introduction of an assessment instrument like the interRAI-LTCF has consequences for the care process at all of these different levels. We studied the consequences at the level of the involved professionals and management. But not only the introduction (initial phase) but also the maintenance is often under pressure in health organisations due to shifting priorities, lack of time and money needed for ongoing and renewed training and equipment [3].

The research questions that are addressed in this paper are:

1. Which factors facilitated or impeded the introduction of the interRAI-LTCF as part of the Multidisciplinary Integrated Care model in residential care homes?

2. Which factors facilitated or impeded the use of the interRAI-LTCF in the maintenance phase, three years after introduction?

## Methods

In this study a mix of quantitative and qualitative methods was used. The study was approved by the ethics committee of the VU University Medical Center in Amsterdam, the Netherlands. All participating residents or their proxies provided written informed consent.

During the *initial phase* of the implementation, the opinions and experiences of all home managers, nursing assistants and family physicians were collected in a survey and structured interviews in 5 out of 10 residential care homes that were randomised to start working with the multidisciplinary integrated care model [6]. At the initial phase we used a survey with fixed answering options which we could compare across the homes (Tables 1, 2).

**Table 1:** **Opinion staff and family physicians of intervention homes** (**n** = **5**) **on multidisciplinary integrated care research question 1**

**Opinion**	**Staff N** = **17**	**Family physicians N** = **14**
Increased expertise after RAI use,%	52.9	54.5
Quality of multidisciplinary meeting increased,%	64.7	81.8
More knowledgeable about resident’s health,%	52.9	63.6
Improved cooperation family physician and staff,%	58.8	81.8

**Table 2:** **Experiences of staff and managers** (**n** = **22**) **of 5 intervention homes in the initial phase research question 1**

**%**	**Yes**	**No**	**No opinion**
**Sufficient introduction and training staff**			
Overall	52.9	29.4	17.6
**Autonomy and self guidance staff**			
More expertise	52.9	35.3	11.8
Improvement quality of care	35.3	17.6	47.1
Enough support	88.2	5.9	5.9
Better overview of health problems	58.8	29.4	11.8
**Time investment and applicability of the interRAI**-**LTCF**			
Enough time available	5.9	76.5	17.6
Sufficient computer equipment	29.4	47.1	47.1 23.5
RAI software is user friendly	70.6	17.6	11.8
**Quality of care**			
More knowledge about health of resident	52.9	35.3	11.8
Earlier detection of health problems	47.1	23.5	29.4
Better discussion of complex care needs in Multidisciplinary Meeting	64.7	17.6	11.8
Family physician is better informed	70.6	17.6	11.8
More and better communication in team	52.9	29.4	17.6

During the *maintenance phase*, three years after the introduction in 10 homes, we selected three homes that differed on their quality of care performance according to quarterly benchmark reports produced for facilities in the Netherlands that work with the interRAI LTCF. We selected the best, the worst and one average scoring home according to the benchmark.

From these three homes the managers (n = 3) and two staff members were interviewed (n = 6). The staff members were nurse assistants who work daily with the interRAI-LTCF and coordinate care planning of the individual residents. We used semi structured interviews to get more in depth insight in impeding and facilitating factors. These interviews were recorded, noted verbatim and interpreted independently by two researchers (MB, EL) (Tables 3, 4, 5, 6).

**Table 3:** **Benefits of the interRAI-LTCF according to staff** (**n** = **6**) **of 3 homes**

**Residential home 1 best scoring home**	**Residential home 2 average scoring home**	**Residential home 3 worst scoring home**
The graphics and the plots in the outcome report show directly if there are improvements or that some conditions have become worse	The graphs and plots are useful in that they show a decline, stabilization or improvement on the physical or cognitive area that is immediately visible	The signalling of issues that you previously paid no or little attention to is very helpful because now it is acknowledged that these were serious issues important to the resident.
All the information on residents is in a database and on the computer		Clear view of the residents actual needs.
MM’s are improved. Better cooperation with all the disciplines involved		It is helpful in that it gives indications for need and utility of care
More standardized methods to work with.		Easy to use.
		First seen as extra workload now the usefulness is seen in, MM with all disciplines and there is more structure in the care process.

MM: Multidisciplinary Meeting.

**Table 4:** Drawbacks of the interRAI-LTCF according to staff (n = 6) of 3 homes

**Residential home 1**	**Residential home 2**	**Residential home 3**
**Best scoring home**	**Average scoring home**	**Worst scoring home**
Too much time spent behind computer instead of providing care.	Not all the important information regarding a resident comes up in the interRAI-LTCF.	Takes too much time sometimes.
Some syndromes are not in the interRAI-LTCF.	It is too limited to see the total condition of a resident.	It does give a little bit extra work.
Sometimes the interRAILTCF gives unnecessary information.	There is still resistance among the employees working with interRAILTCF because we do not see the additional value.	
Not all the important information comes up in the interRAI-.LTCF	t is only a guideline, it could be improved to better fit the needs of the people on the work floor who actually have to fill out the interRAILTCF.	
	Some sections in the interRAI-LTCF have not enough/ or the right questions to get a adequate overview of the resident.	
Good observing and communicating with residents and their relatives and the physician providesalso a good picture of the care a client needs.	There always needs to be oral explanation in a Multidisciplinary Meeting otherwise not all information and problems become clear.	
The interRAI-LTCF does not give any extra value to the care plan.	It takes to much time to fill in the interRAI-LTCF.	
interRAI-LTCF is now accepted but only because it is made mandatory.	There is too much standardization in using the interRAI-LTCF.	

**Table 5:** **Benefits according to the management** (**n** = **3**) **of 3 homes during maintenance phase**

**Residential home 1**	**Residential home 2**	**Residential home 3**
**Best scoring home**	**Average scoring home**	**Worst scoring home**
Nursing assistants are more aware of the specific care needs because the care model considers the case history of the resident.	One system. Consistency in methods. Everyone uses the same standards.	More awareness is created of the specific care needs.
It creates awareness of improvements that can be made in the care process. The structure of the care giving process has improved.	Communication is easier because one set of terms is used.	More attention to the wishes of clients.
Care plans can be created with the specific care a resident needs and his or her wishes and preferences.	Deeper insight in the resident’s needs and wishes.	
The new care model has led to more efficient use of personnel. The interRAILTCF gives a clear overview of the hours of care that are needed on the residents and the wards.		

**Table 6:** **Drawbacks according to the managers** (**n** = **3**) **of 3 homes during the maintenance phase**

**Residential home 1**	**Residential home 2**	**Residential home 3**
**Best scoring home**	**Average scoring home**	**Worst scoring home**
InterRAI-LTCF sometimes cannot detect specific syndromes	Time consuming.	Questions are too complicated
Sometimes interRAI-LTCF gives a trigger but it is not always clear if something needs to be done and what needs to be done.	The system is not always available.	Not completely in line with the care need indication
The translation of the outcomes is sometimes difficult to link to specific actions.	Sometimes difficult to interpret 6 monthly outcome reports.	The instrument needs revising for better use in practice
Sometimes the interRAILTCF system is not working on the computers	Not all medication is available in the system.	Scores on the benchmark reports are easily distorted when there are in practice only one or two worse cases in the facility
Outcomes of the benchmark reports are difficult to point to the specific residents.		The personal factor must be kept in mind. Not only focusing on the instrument itself, but listening and looking at the resident as well.

### Multidisciplinary integrated care model

The Multidisciplinary Integrated Care model was inspired by the chronic care model. Assessment with interRAI-LTCF of the patient’s functional health and care needs is imperative. This enabled immediate identification of problem areas which supports individualised care planning. Secondly, the assessment outcomes were discussed in a Multidisciplinary Meeting (MM) with the nursing assistant, family physician, elderly care physician and psychotherapist. The Multidisciplinary Meeting (MM) provided advice on management and treatment of modifiable disabilities and risk factors. Thirdly, consultation by a geriatrician and psychotherapist was offered to the frailest residents at risk for nursing home admission. Finally, a quarterly benchmark report compared 32 risk adjusted indicators of quality of care across all residential and nursing homes in the Netherlands that used the interRAI-LTCF [9,10]. Management can use this overview to improve specific areas of care. For example, if the (case mix adjusted) number of falls is substantially higher compared to the expected average (benchmark), management can decide on measures to improve safety in a particular home. The Association of RAI users in the Netherlands, Nedrai, owns the software, and provides these overviews for a limited tariff per resident (http://www.nedrai.nl).

### The initial phase of the intervention

In the initial phase 45 nursing assistants, five team coaches and managers of five residential care homes were trained in a two day course by external trainers. The coaches and nurse assistants of each home were trained together. The managers were trained separately in using the management output and quality indicators.

In this course the background and position of the assessment instrument in the Multidisciplinary Integrated Care model was explained and the use of the web based version of the interRAI-LTCF was practiced. Special attention was given to designing individualized care-plans based on the assessments and in sharing this information with the residents or their relatives and other professionals like the family physician. The care organisation appointed a project leader during the implementation for two days a week. A steering committee was installed that initially met two weekly and less frequently as the project enrolled. In the maintenance phase new staff received in company training as sufficient experience was gained within the organisation.

### Measurements and data analyses

#### For research question 1

Which factors facilitated or impeded the introduction of the interRAI-LTCF as part of the Multidisciplinary Integrated Care model in residential care homes?

Structured interviews were held in the initial phase of the introduction of the care model and interRAI-LTCF with nursing assistants, team coaches and managers of the intervention homes randomised in the randomised clinical trial study. In addition, a brief questionnaire was send to the family physicians who were involved. The interview and the questionnaire were composed based on literature review, expert opinion and piloted before the data collection. The questions covered subjects like: training aspects, autonomy and self guidance for staff, time investment and applicability of interRAI-LTCF, quality of care, and communication between family physicians and staff. Those interviews were held and analysed by one researcher and supervised by two other researchers. The answers of the questionnaire are presented in percentages (Tables 1, 2).

#### For research question 2

Which factors facilitated or impeded the use of the interRAI-LTCF in the maintenance phase, three years after introduction?

The information and data of the maintenance phase was collected through in-depth interviews with three home managers and six nurse assistants who used the instrument. The focus of these interviews was to elicit opinions on the instrument and its use in real life work. The managers were chosen to be interviewed because of the overview they have in the homes and the possible usefulness of the interRAI-LTCF according to their perspective.

From each of the facilities two staff members were interviewed. These staff members were nurse assistants who worked on a daily base with the interRAI-LTCF.

To obtain sufficient background information several interviews were held with the two project managers initially responsible for the implementation of interRAI-LTCF in the homes. During the interviews the conversations were taped and described verbatim afterwards.

During the analysis these notes could be re-read and the conversations re-listened to make sure all was noted well [11]. The data collected in the interviews was displayed in tables (Tables 3, 4, 5, 6, 7).

**Table 7:** **Impeding and facilitating factors during maintenance according to managers** (**n** = **3**) **and staff** (**n** = **6**) **of the best**, **the worst and an about average scoring home according interRAI-LTCF outcomes** (**n** = **3**)

**Residential care homes**	**Score on the outcome reports**	**interRAI LTCF implemented in**	**Resistance at introduction**	**Resistance in the present**
Nr 1	The best	2007	Management: yes	Management: no
			Care team: yes	Care team: no
Nr 2	Average	2007	Management: yes	Management: no
			Care team: yes	Care team: yes
Nr 3	Worst	2008	Management: no	Management: no
			Care team: no	Care team: no

## Results

### Context at initial phase

The average size of the homes was 46 residents. The average ratio nursing assistant to residents was 1 to 15. Staff was predominantly female (88.2%) had a mean age 41.4 years, and the majority (64.7%) completed an education of nursing assistant and 17.6% of nurse. They had a mean job experience of 17.9 years and the average work hours a week was 28.4 (sd 4.4).

Resident mean age was 85.8 (sd 7), 24.3% was male, 55.4% was cognitive impaired, and the mean number of medication was 7.6 (sd 3.5).

The intervention homes scored better on 30 of the 32 risk indicators for quality of care and of these had improved significantly [16]. Most notably, bowel incontinence decline dropped from 23 to 6%, bladder incontinence decline dropped from 46 to 15%, delirium risk dropped from 56 to 28%, pain worsening dropped from 41 to 13%. The intervention homes performed also significantly better when the sum scores were compared. The residents in the intervention homes tended to be more satisfied about the quality of care than elderly in the control homes.

### Staff and family physicians' opinions

Seventeen nursing assistants, five team coaches and three managers of 5 residential care homes as well as 14 family physicians were interviewed (Tables 1, 2). The majority (82.4%) of the nursing assistants was satisfied with the interRAI-LTCF and 58.8% considered the individual parts of the instrument as obvious. 47.1% was positive about the training courses. But the same percentages want more explanation over the usefulness of the instrument. Staff was also satisfied of the applicability of the software supporting the interRAI-LTCF assessments (71%). About 55% of the family physicians (n = 14) considered the quality of care to be improved. They acknowledged that there was a need for a new care model (73%). 52.9% of the nursing assistants had the opinion that their competence had increased with the application of interRAI-LTCF but only 35.3% considers the quality of care improved. The managers were divided on this point. They say that the use of interRAI-LTCF introduced uniformity and the possibility of quality control.

### Impeding factors according to nurse assistants and managers

Not enough time to fill in the interRAI-LTCF (76.5%), not enough computer equipment (47.1%) and insufficient computer equipment (64.7%) were reported by personnel.

### Context maintenance phase 3 years after introduction

All residential care homes used interRAI-LTCF assessments for their residents (n = 426) every 6 months. In 2007 the interRAI-LTCF was implemented in the best and the average performing home, and in 2008 in the worst scoring home. The latter home faced problems like frequent change of management and lack of nursing staff (Tables 3, 4, 5, 6, 7).

### Resistance to change

Staff in all three facilities still tended to have resistance against working with the interRAI-LTCF. Only in home Nr 3 (the worst scoring home) staff was less resistant. Management in all three researched facilities described that they tried to reduce this resistance by emphasizing the usefulness of this new assessment system and involving staff by taking courses in the use of the interRAI-LTCF.

### Facilitating factors

The majority of nursing assistants felt that the instrument facilitates better communication with colleagues and other disciplines like the family physicians and specialists of elderly care. Nursing assistants do mention that they have a better overview of the health problems of the residents and mention improvement of the quality of Multidisciplinary Meetings with the use of the interRAI-LTCF. Impeding factors after 3 years were the lack of time and insufficient computer equipment. Some nursing assistants claimed that the benefits of the interRAI-LTCF were not clear to them. Overall, in content and quantity the nurse assistants of Residential Home 2 were least positive about the use of the interRAI-LTCF. Remarkably, nursing assistants of the ’worst’ scoring residential home saw the least drawbacks and the most benefits of the interRAI-LTCF. This is somewhat contradictory to the finding that they scored the ‘worst’ compared to the other two homes on the quality of care outcome report. This may be explained by the fact that this particular home faced management disruption. The new manager tended to be very positive about the instrument and the care model.

## Discussion

Resistance to change is an issue occurring in all organizations when change is required. Many people working in health care are not interested in change or feel the need to change. Management in all three researched facilities described that they tried to diminish this resistance by communicating the usefulness of this new assessment system and involving staff by taking courses in the use of the interRAI-LTCF. They all feel that there is no longer any resistance now. The manager of residential care home 1 mentioned that during the introduction of the interRAI-LTCF assessment a lot of changes in management and financial support were on going in the organization. This was the main reason for some aversion to this ‘new management thing’. In order to deliver a better quality of care availability of sufficient staff and resources as computer equipment is obvious [17]. Based on the opinions of staff different signals were found. In RH 1 nursing assistants feel that there are in general not enough people working in the organization and that there is not enough staff that can fill in the interRAI-LTCF in the appropriate manner. In RH 2 and 3 sufficient staff is available to fill in the interRAI-LTCF according to the nursing assistants. *Time* is, as comes clear, another important resource. In all residential care homes four hours a week are scheduled to fill in the interRAI-LTCF. According to the managers more time could be needed for appropriate use of the interRAI-LTCF. In RH 2 and 3 staff mentioned that there is sufficient time available to work with the interRAI-LTCF, but staff in RH 1 felt that there is not enough time available.

Management and staff of all three facilities agree that the computer equipment is not sufficient to work with the interRAI-LTCF. The manager of RH 3 brought up the suggestion to use laptops in the future. The interRAI-LTCF can be filled in together with the resident and family in the residents’ apartment. Doing so involves residents and family in the care process. *Teamwork* can be seen as inter and intra organizational cooperation between organizations and people. The output of the interRAI-LTCF on residents will lead to shared decision making and involves members of the multidisciplinary team in the steps to be taken in the individual care process. Complex care needs will be discussed in the Multidisciplinary Meeting (MM). All interviewee’s feel that using the interRAI-LTCF improves teamwork by better information, enables better structuring of MM’s and shared decisions on the care plan. The culture of the facilities has changed by using the interRAI-LTCF. In most health care organizations there is an unofficial hierarchy where nurses, or nurse assistants will and cannot easily tell their opinion to a physician [14]. With the use of the output of interRAI-LTCF as basic information guiding the care process and structuring the MM all care givers need to work together to create the best possible care for the resident. Opinions of nurses and nurse assistants are appreciated and taken into account. As family physicians mention that residents in residential care homes need more complex care and that the availability of skilled staff is under pressure empowering of the nursing assistants will be important. Training and empowerment of nursing assistants together with monitoring using the interRAI-LTCF were likely to be the most important ingredients for improvement of the quality of care [16]. Statement of RH 2 manager: *‘RAI has created a deeper insight in the resident’s needs and wishes.*

### Comparison with literature

There is to our knowledge no literature on implementation of a care model using interRAI-LTCF in residential care homes. In several studies about staff satisfaction using a Resident Assessment Instrument version in the US [8,13] was found that more than 64% of nursing home directors felt that it is worth time and effort spent by staff. They also felt that the quality of care planning and the assessment of patients improved in comparison to other instruments they were using before.

The opinion of management in our study on improvement of quality of care by using the interRAI-LTCF is a full outspoken yes. This is in line with the results of other studies [7,18]. Patient assessment by means of the interRAI-LTCF provides more accurate information about the needs and wishes of patients than single assessment instruments. Client- tailored care plans can be formulated on the basis of this detailed multiple domain information. These client- tailored care plans can diminish the gap between patient’s needs and expectations of the care they need and the actual care provided. Consequently, this will lead to improved quality of care [17]. Staff and management in our study also mentioned a better understanding of the needs and wishes of the residents. In another study [18] the interRAI-LTCF was described by 73% of the interviewed nursing assistants as a tool able to give a ’whole picture ‘of the resident, allowing the nursing assistants to ’know the resident better’, and a practical instrument for providing better care to residents. Like Bernabei we also found resistance likely due to the ignorance of the rationale of the multidimensional assessments by people working in geriatric care. Various professionals like the geriatrician and psychotherapist and some nursing assistants had a hard time accepting the rationale of such an assessment tool and system. Other impeding factors like staffing problems and inadequate equipment were also found by Holtkamp (2003) and Achterberg (2004) [17,19].

### Strengths and limitations

We were able to achieve participation from a large and extremely difficult study population and implemented the Multidisciplinary Integrated Care model in a single care organization having the possibility of uniform implementation. This contributed greatly to the internal validity of the study results. The introduction phase took 3 months including training of the nurse assistants in using interRAI-LTCF. After the 6 month period of the main study the care model including working with interRAI-LTCF was implemented in the control homes within 3 months. A project manager of the care organization provided in the introduction as well as in the maintenance phase coaching on the job of the nurse-assistants and the managers.

There are some limitations. The cluster randomization produced an imbalance between the intervention and control homes in the number of participating residents and in some of the functional characteristics of the residents at baseline. Although we adjusted for the imbalance in functional characteristics, imbalance in the number of participating residents may have led to underpowered results. Variation across the intervention homes in the application of the complete protocol (3%–66%) was another limitation of the main study. This variation can be explained by financial and administrative issues during the study period of the main study. The financial obligations for residential care homes resulting from a new national funding system for residential care of elderly people caused uncertainty about job continuation, high turn over of managers, and new priorities of the homes in our study. Only 55.2% percent of the residents in the intervention homes were assessed with the Long Term Care Facility version during the study period of 6 months. This was less than we aimed for and was partly due to implementation delay [16]. For example, one intervention home actually started RAI assessments after 6 months because the house manager was on sick leave.

For the first research question the investigated sample was limited due to lack of time, illness and vacation of managers and nursing assistants. In addition, the interviews used in the initial phase were tested on construct and expert validity but not on criterion validity and reproducibility.

For the second research question an important limitation was the relatively small sample which was due to the exploratory and in- depth nature of the interviews. Another possible limitation is that this study was conducted in three residential care homes all belonging to the same care organization. Because of the small sample and the exploratory nature of the research no real generalization can be made.

## Conclusions

The main conclusion of this study is that the facilitating and impeding factors in the initial phase as well as in the continuing phase were basically the same.

Facilitating factors as positive opinions and support of managers is in both phases of great importance. Our findings are in line with the conclusion of Grimshaw that the implementation of clinical guidelines within a clinical governance setting requires time, enthusiasm, and resources.

Pointing out the benefits of the new care model with the use of a time consuming assessment is an important factor for staff in the initial phase. These benefits of the assessment tool for nursing assistants are a better understanding of the clients' problems, enabling them to go beyond merely executing physicians' orders and to become active players in planning clients' care.

The impeding factors as shortage of time and insufficient equipment that we found in both phases which frustrated the process may be typical for the Dutch circumstances in long term care facilities.

### Practical implications

Although the benefits seem to overrule the drawbacks, implementing a new care model by using an assessment tool like the interRAI-LTCF is not an easy process. But the randomized clinical trial in which this implementation was initiated demonstrated a substantial increase in quality of care [16]. Like other studies we also found a positive effect on the role of the nursing assistant in coordinating and guiding the care process [7,17-20].

The results of this study are not only applicable in residential and nursing home care but also for community dwelling elderly.

## Abbreviations

InterRAI-LTCF: Internet application of resident assessment instrument long term care facility version; RAI-HC: Home care version of the resident assessment instrument; MM: Multidisciplinary meeting.

## Competing interests

The authors declare that they have no competing interests.

## Authors' contributions

MB, EL and HvH drafted the paper. TE, DF, GN critically commented on the draft and all authors approved the final version.

## Pre-publication history

The pre-publication history for this paper can be accessed here:

http://www.biomedcentral.com/1472-6963/13/8/prepub

## Supplementary Material

Additional file 1 **Figure S1.** Difference between usual care group and intervention group scores on 24 'mother' risk indicators of quality of care. Click here for file
