# Supplementary material for: Implementation of geriatric assessment and decision support in residential care homes: facilitating and impeding factors during initial and maintenance phase
Source: BMC Health Serv Res. 2013 Jan 5;13:8. doi: 10.1186/1472-6963-13-8 (PMC3601009; doi:10.1186/1472-6963-13-8)

Figure S1. Difference between usual care group and intervention group scores on 24 'mother' risk indicators of quality of care,

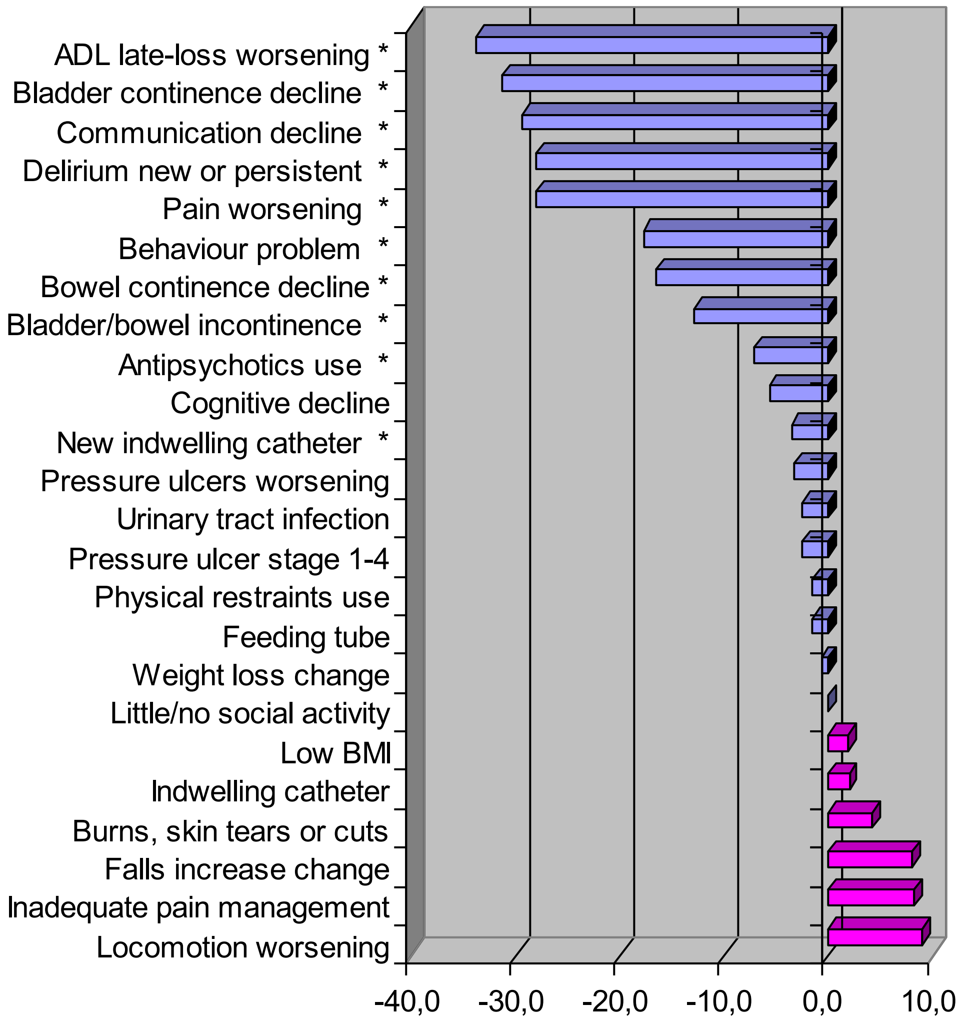

Supplement: Additional file 1 — Figure S1. Difference between usual care group and intervention group scores on 24 'mother' risk indicators of quality of care. [file 1472-6963-13-8-S1.pdf]
